# Self-confidence and knowledge of suicide assessment and prevention amongst first-line health professionals in Nelson Mandela Bay, South Africa

**DOI:** 10.4102/safp.v64i1.5377

**Published:** 2022-01-10

**Authors:** Adeyinka A. Alabi

**Affiliations:** 1 Department of Family Medicine and Rural Health, Dora Nginza Regional Hospital, Gqeberha, South Africa; 2 Department of Family Medicine and Rural Health, Faculty of Health Sciences, Walter Sisulu University, Mthatha, South Africa

**Keywords:** suicide, suicide attempts, first-line health professionals, knowledge, competency

## Abstract

**Background:**

First-line health professionals are uniquely positioned to recognise suicidal behaviours in patients. However, the opportunities are often missed or poorly managed. Self-confidence and knowledge of suicide prevention and assessment by health professionals can lead to prompt recognition and management of at-risk individuals. This study evaluates the first-line health professionals’ self-confidence and knowledge of suicide assessment in Nelson Mandela Bay Municipality (NMBM), South Africa.

**Methods:**

A cross-sectional study was conducted in six healthcare facilities across NMBM between January 2020 and March 2020. Five hundred first-line healthcare professionals were recruited to respond to a validated self-administered questionnaire to collect demographic characteristics, self-confidence levels and knowledge of suicide assessment and associated factors.

**Results:**

A total of 344 first-line health professionals completed the questionnaire (68.8% response rate); 40% of the respondents work in emergency units and 77.3% reported frequent encounters with patients who attempted suicide. Most participants had not received suicide assessment training during their undergraduate or postgraduate years (59.6% and 81.1%, respectively). They also lacked adequate knowledge and self-confidence in suicide assessment. Younger age, minimal work experience and attendance of two or more hours of suicide prevention training were associated with higher knowledge of suicide assessment.

**Conclusion:**

Findings revealed gaps in self-confidence and knowledge of suicide management, attributed to lack of training in suicide management. Health authorities should prioritise upskilling of front-line workers in suicide prevention and assessment, specifically targeting older nurses in the region.

## Introduction

Worldwide, suicidal behaviour is a major public health concern, with an estimated 800 000 people having died by suicide and many more attempting suicide every year.^1^ The low-income and moderate-income countries carried the brunt of the problem with over 75% of the suicide deaths.^2^ Suicide is the end result of distressing complex interaction between biological, psychological and social crises, and is a preventable cause of deaths.^3^ Evidence-based effective preventative strategies include restriction of access to means of suicide, regular training of healthcare professionals in suicide prevention, increasing public awareness and optimal care of those with mental disorders.^4,5^

The World Health Organization (2012) identifies suicide as a preventable death if all the stakeholders play their role.^6^ In the majority of persons who died by suicide, there had been windows of opportunity open to first-line healthcare workers to interrupt the suicidal process but these were often missed or improperly managed.^7^ First-line healthcare professionals (nurses, general practitioners, family physicians, emergency department staff and clinical associates) are well placed to assist persons with occult suicidal behaviour through routine screening at the primary health centres. Many of the patients with ongoing suicidal behaviour present to first-line healthcare professionals with other complaints, without disclosing the suicidal intent.^8,9,10^ First-line healthcare professionals need to possess a good clinical knowledge of suicide assessment to be able to identify patients with undisclosed ongoing suicidal behaviour, failure of which could result in missing patients planning imminent suicide.^11,12^

Furthermore, inadequate screening for depression and other mental illnesses by first-line healthcare professionals at the primary health care (PHC) level results in unrecognised and untreated mental illness.^13,14,15^ The volume of work, non-availability of human and material resources and inadequate knowledge of healthcare professionals have been cited as contributory reasons for the poor detection and screening conducted by healthcare professionals at PHC facilities.^16^ Despite the devastating consequences of suicide on families, the health status and economy of a nation, there has been a paucity of published data on first-line healthcare professionals’ knowledge and self-confidence in assessment of patients with suicidal behaviour in the Eastern Cape, South Africa. This study aimed to evaluate the first-line healthcare professionals’ knowledge and self-confidence in conducting suicide assessments. The study objectives were to (1) evaluate first-line healthcare professional’s knowledge of risk factors and warning signs of suicide (2) assess the self-confidence of first-line healthcare professionals in assessment and management of patients with suicidal behaviour.

## Method

### Study design and setting

A multicentre cross-sectional study was conducted between 15 January 2020 and 15 March 2020 at health facilities in the Nelson Mandela Bay Municipality (NMBM) of South Africa. The municipality is situated in the western region of the Eastern Cape Province and consists of three urban cities; Gqeberha, Despatch and Uitenhage. The health structure in the municipality consists of three provincial hospitals, and 48 PHCs, out of which seven are community health centres (CHCs). Purposive sampling of six health facilities: two hospitals (Uitenhage provincial hospital and Dora Nginza regional hospital), two CHCs (Motherwell Health and Letitia Bam) and two PHCs (Rosedale and Mission vale) was conducted. The researcher’s choice for the two provincial hospitals was because they offer primary and secondary level mental healthcare services. The CHCs and PHCs were selected based on the drainage areas of the two provincial hospitals. Dora Nginza hospital has 35-bed designated mental health unit and a 24-bed 72-hour mental health observation unit (72-hour unit). The designated unit has a full complement of psychiatrists, psychiatric registrars, clinical social workers, occupational therapists and clinical psychologists. The 72-hour unit is under the headship of family physician with medical officers, a psychologist and psychiatric nurses. The portal of entry for all mental healthcare users needing in-patient admission is casualty, where relevant medical workup is completed before onward transfer to the 72-hour unit. The mental healthcare services offered in the hospital include acute psychiatry, chronic inpatient care, outpatient clinics, liaison-consultation and rehabilitative services. Uitenhage provincial hospital has a 10-bed acute psychiatry ward and two of the feeder clinics were also selected as study sites. The four selected primary healthcare centres are being run by psychiatric nurses with a roving medical officer who visits each clinic once a week on a rotatory arrangement. Patients needing hospitalisation from the CHCs and PHCs are discussed and transferred to the provincial hospital of their drainage area.

### Sampling technique

A targeted sample of 500 healthcare professionals was recruited from the six health facilities, from which 344 healthcare professionals completed the questionnaires (68.8%). Participants were included in the study if they met the following criteria: 18 years and older, work in units with direct contact with patients who presented with suicidal behaviour and willing to participate in the study. Three trained research assistants distributed the questionnaire to be self-administered.

### Data collection and measures

After obtaining informed consent, each participant completed a self-administered questionnaire. This questionnaire, which was previously validated in other studies, was used to collect data on demographics, knowledge and self-competence in suicide assessment.^17,18^ Participants were asked to rate their knowledge of suicide prevention on this seven-item questionnaire. Self-rating was carried out by ticking blocks on a Likert scale ranging from 1 (very low) to 5 (very high). Although all the items in the instrument were previously validated in other population, pilot study was conducted with the instruments amongst 10 first-line healthcare professionals in one of the study sites. Minor adjustments were made after reviewing the responses from the participants in the study. The results of the pilot study were excluded from the main study.

A more objective knowledge of suicide assessment was evaluated with eight-item agree or disagree questions, where the responses were scored as either correct or incorrect. The maximum and minimum obtainable scores of 0 and 8, respectively, indicated that those with higher scores had greater knowledge.

The confidence of participants in assessment and management of patients with suicidal behaviour was assessed with three-item questions rated on a 5-point Likert scale (1 = strongly disagree, to 5 = strongly agree). A minimum and maximum obtainable score of 1 and 15, respectively, indicated that those with higher score had greater confidence.

### Data analysis

Participants’ responses were coded and entered into Epi info version 3.5.3 software. Data analysis was performed using STATA software, version 15.0 (Stata Corporation, College Station, TX, United States [US]). Descriptive analyses (frequencies, percentage and standard deviations) were used to describe the demographic characteristics and to assess the level of knowledge and self-efficacy in suicide assessment. Chi-square test and bivariate analysis were used to assess associations between knowledge and independent variables. Variables that were significant at *p* < 0.05 level were reported for factors associated with level of knowledge in suicide assessment and prevention.

## Results

A total of 500 questionnaires were administered and 344 participants returned the questionnaires, thus, giving a response rate of 68.8%. The participants comprised 216 nurses (62.8%), 108 medical practitioners (31.4%), 11 psychologists (3.2%) and nine social workers (2.6%). The majority of the participants were female (*n* = 246, 71.5%) and 196 were 30 years and below (57.0%). The majority of the participants reported not having received any training in suicide prevention during their undergraduate training (*n* = 205; 59.6%). Furthermore, most of the participants had not received any training on suicide prevention, post-graduation (*n* = 279; 81.1%). More than half (*n* = 189; 54.9%) of the participants reported having seen between 1 and 10 patients with suicidal behaviour in the previous month (Table 1).

**TABLE 1:** Background characteristics of healthcare workers included in the study.

Variables	Frequency count	Percentage
**Age**		
30 years or less	165	48.0
31–40 years	95	27.6
41–50 years	52	15.1
51–60 years	32	9.3
**Gender**		
Male	98	28.5
Female	246	71.5
**Home language**		
English	65	18.9
Afrikaans	63	18.3
Xhosa	199	57.8
Others	17	4.9
**Profession**		
Medical doctor	108	31.4
Nurse	216	62.8
Psychologist	11	3.2
Social worker	9	2.6
**Year of experience**		
1–2 years	104	30.2
3–5 years	76	22.1
6–10 years	75	21.8
> 10 years	89	25.9
**Workplace**		
Clinics	105	30.5
Dora Nginza	188	54.7
Uitenhage Hospital	51	14.8
**Unit of primary assignment**		
72-h ward	33	9.6
Psychiatry ward	22	6.4
Casualty	136	39.5
Medical ward	71	20.6
Maternity	61	17.7
Not stated	21	6.1
**Duration of undergraduate training in suicide assessment**		
None	205	59.6
0–1 h	40	11.6
2–3 h	70	20.3
4 h and more	29	8.4
**Duration of postgraduate training in suicide assessment**		
None	279	81.1
0–1 h	27	7.8
2–3 h	20	5.8
4 h and more	18	5.2
**Number of suicide patients seen in the past year**		
Zero	78	22.7
1–10	149	43.3
11–20	39	11.3
21–30	41	11.9
31 and more	37	10.8
**Number of SP seen last month**		
Zero	142	41.3
1–10	189	54.9
11 and more	13	3.8

SP, suicidal behaviour patients.

### Knowledge of suicide assessment and prevention

The result showed significant group differences in the mean total knowledge score with medical doctors having the highest score (mean 4.95) followed by social workers with an average score of 4.89. Nurses had the lowest mean score of 3.77 (Table 2). Less than 40.0% of the participants rated themselves highly and very highly in the knowledge of suicide and prevention. About one-third (29.9%) of the participants rated their general understanding about suicide prevention as ‘high’ or very ‘high’. Only 32.6% of the participants rated themselves as high or very high on how to ask someone if they have the feeling of committing suicide (Table 2).

**TABLE 2:** Association between background characteristics and knowledge of suicide.

Interdependent variable	Frequency	Average Knowledge scores (s.d.)	s.d.	*p*-value
**Age**				
30 years or less	165	4.35	1.38	0.053
31–40 years	95	4.03	1.12	
41–50 years	52	3.88	1.28	
51–60 years	32	4.44	1.58	
**Gender**				
Male	98	4.43	1.31	0.044
Female	246	4.11	1.32	
**Qualifications**				
Medical doctor	108	4.95	1.20	< 0.001
Nurse	216	3.77	1.22	
**Year of experience**				
1–2 years	104	4.47	1.17	0.072
3–5 years	76	4.14	1.37	
6–10 years	75	3.97	1.35	
> 10 years	89	4.12	1.40	
**Duration of undergraduate training in SP**				
None	205	3.84	1.22	< 0.001
0–1 h	40	4.55	1.32	
2–3 h	70	4.89	1.15	
4 h and more	29	4.62	1.59	
**Duration of postgraduate training in suicide assessment**				
None	279	4.06	1.30	0.001
0–1 h	27	4.70	1.17	
2–3 h	20	5.0	1.34	
4 h and more	18	-	1.46	

SP, suicidal behaviour patients; s.d., standard deviation.

However, just half of the sample performed well in the objective assessment of knowledge of suicide risk factors and warning signs. The first statement requiring either agreement or disagreement reads, ‘People who talk about suicide do not kill themselves’ to which 41% of the participants answered incorrectly. The correct response should have been to disagree. Statement two, requiring the same affirmation or rejection stated:

‘[*P*]eople who make plans to commit suicide keep their thoughts to themselves to which 68% of the sample answered incorrectly. The correct answer should once again have been to disagree. The third statement reads, ‘everyone who commits suicide is depressed’.

The correct response should have been to disagree. The fourth statement reads, ‘Suicide tendencies are inherited, and suicide runs in families’ and 39.8% of the participants answered incorrectly. The correct response should have been to disagree. The fifth statement, ‘Removing the means of suicide would prevent many suicides’ drew an incorrect answer from 48.5% of the participants. The correct response should have been to agree. The sixth statement, ‘There are one or two causes or motives that explain most suicides’ evoked an incorrect answer from 41% of the participants. The correct response should have been to agree. The seventh statement read, ‘There is no relationship between drugs/alcohol and suicide’ and 33.4% of the participants answered incorrectly. The correct response should have been to disagree. The eighth statement, ‘Suicide is always the action of a patient suffering from a mental- or psychotic illness’. To this, 43% of the participants answered incorrectly. It would have been correct to have disagreed. The operative word in the statement was ‘always’ and this could not have been proven empirically.

### Association between baseline characteristics and knowledge of suicide prevention

As shown in Table 2, participants aged 30 years or less, male gender, being medical doctors and having attended two or more hours of training whether in undergraduate or post-graduate were associated with high knowledge of suicide prevention and assessment.

### Self-confidence in suicide assessment

Out of all the participants, 105 confirmed that they were hesitant to ask a patient if he or she is having suicidal tendency (30.5%). About 40.0% of the participants reported being confident in their ability to successfully assess patients with suicidal behaviour and fewer participants (32.5%) were confident in the management of patients with suicidal behaviour (32.5%) (Table 3).

**TABLE 3:** Self-competency to treat suicidal behaviour patients.

Items	Strongly disagree		Disagree		Undecided		Agree		Strongly agree	
	*n*	%	*n*	%	*n*	%	*n*	%	*n*	%
I am confident in my ability to successfully assess suicidal patients	47	13.7	72	20.9	86	25.0	111	32.3	28	8.1
I am confident in my ability to successfully treat suicidal patients	51	14.8	99	28.8	82	23.8	95	27.6	17	4.9
I am hesitant to ask patients if he or she is having suicidal behaviour	54	15.7	128	37.2	57	16.6	88	25.6	17	4.9

## Discussion

Given the unique position of the front-line healthcare professionals in encountering individuals with suicidal behaviour at the healthcare facilities, missed opportunities to detect and manage people who may have suicidal ideations and plans may lead to catastrophic outcomes. This study seeks to assess the knowledge and self-confidence of first-line healthcare professionals in the assessment and management of suicide. This study shows that the majority of first-line healthcare professionals do not have sufficient knowledge of suicide assessment. The mean score in the objective assessment of knowledge was only half of the total obtainable score (4.59). Whilst medical doctors (4.95) and social workers attained just above half of the total obtainable score, nurses attained less than half of the total obtainable (3.77). In the self-rating of knowledge of suicide assessment and management, less than 30% of the participants rated themselves ‘highly’ or ‘very highly’. Furthermore, more than half of the participants did not have confidence in assessing and managing patients with suicidal behaviour. These findings raise a great deal of concern, bearing in mind the cardinal role of first-line healthcare professionals in suicide prevention.

Our findings are in tandem with what was reported in a Kenyan study where nurses working in an emergency unit felt that they had inadequate knowledge and lacked confidence to assess patients with suicidal behaviour.^19^ Our findings are also consistent with a study conducted in the United States where more than 60% of the healthcare professionals felt incompetent and unskilled in suicide risk assessment.^20^ Medical practitioners and social workers had higher mean scores in the objective assessment of knowledge of suicide than nurses did. These findings are consistent with the result of Crawford et al.,^21^ in London, where non-psychiatry doctors had greater knowledge of suicide prevention than non-psychiatry nurses. A plausible explanation for this could be lesser involvement of nurses in suicide assessment. Another likely cause of nurses having the least mean score could be the lower attendance of suicide prevention education amongst nurses than medical doctors. Therefore, to achieve a reduction in suicide rates, there is also a need to capacitate nurses in the area of suicide assessment and management.

In this study, the majority of the participants reported having had regular contact with patients with suicidal behaviour. However, only about 40% of the participants had received training in suicide assessment during undergraduate periods. This finding is consistent with Saini et al.^22^ where only 33% of the primary healthcare professionals had received training in suicide assessment. The low level of knowledge of suicide assessment amongst participants in our study could be attributed to a lack of training in suicide assessment during and after their professional training. Further study will be needed to appraise the curriculum of nursing and medical schools to establish whether there is adequate coverage of suicide assessment and management.

The level of knowledge of suicide assessment was higher amongst those professionals who had two or more hours of training in suicide prevention in our study. This finding is consistent with previous studies which demonstrated an increase in healthcare professionals’ knowledge of suicide assessment after attending suicide prevention training^22,23,24^

In this study, being a healthcare professional aged 30 years or less, with a shorter period of employment, yielded that this group had higher levels of knowledge of suicide assessment than older professionals did. Our result is consistent with that which has been reported in previous studies.^25,26^ The higher mean level of knowledge of suicide assessment amongst younger professionals could be attributed to increased public health concerns of suicide in recent times and possibly more time having been dedicated to suicide prevention during the younger professionals’ training.

## Study limitations

Many health professionals did not return the questionnaire and could not be followed up during the national lockdown because of COVID-19 pandemic. Self-reported knowledge and self-confidence may not necessarily represent a true reflection of the participants’ practice and could be affected by social desirability bias. However, anonymous collection of data allowed the participants to provide their candid responses.

## Conclusion and recommendation

The study shows a low level of self-confidence and knowledge of suicide assessment amongst healthcare professionals working in the health institutions in Nelson Mandela Municipality, Eastern Cape. The study further demonstrated a significant association between attendance of training in suicide prevention and knowledge of suicide assessment. It is, therefore, imperative to have a planned, scheduled in-service training for healthcare professionals in suicide assessment and management at their workstations. This would empower the professionals without disrupting their service delivery.

Improving the staff strength of mental health units of the provincial hospitals will also allow outreaching of clinicians to various PHCs to assist in capacity building of staff at the PHCs. Staff at various facilities should also be encouraged to attend some online educational continuing professional development (CPD) courses that focus on recognition and management of patients with suicidal behaviour. Provision of suicide educational posters, information booklets and standardised guidelines on suicide risks, step wise management, admission criteria and referral pathways should be made available at the workstations of all healthcare facilities in the municipality.
